# Connective Mourning: The Case of Mourning and Memorialization Practices on X

**DOI:** 10.1177/00302228231208112

**Published:** 2023-10-20

**Authors:** Temple Uwalaka

**Affiliations:** 1 School of Arts and Communication, 273718 University of Canberra, Canberra, ACT, Australia

**Keywords:** connective mourning, crisis network theory, networked mourning, online mourning, social media mourning, Queen Elizabeth II, X

## Abstract

This study offers a conceptual framework of connective mourning. The case of mourning and memorialization practices on X. The study demonstrates the crucially different memorialization, and mourning practices and the various resulting parasocial practices and dynamics that they enable. Using the mourning and memorialization of Queen Elizabeth II as a case study, the study point to an emerging practice where through high centrality and density of reciprocity, and low modularity, mourners on X stimulate commonality via decentralized and loose networks that allow for solidarity building during crisis such as mourning. On the Queen specifically, the study grouped those that posted about her death into four categories: *the Grievers*, *the Lauders*, *the Accusers*, and *the Defenders*. This study concludes that when collective mourning occurs, individuals have more reciprocal relationships on a dyadic level which decreases modularity of the network.

## Introduction

The social functions of grief and mourning create social solidarity. These mourning rituals allow mourners to show their love and respect to the deceased (Navon & Noy, 2023; Wagoner & de Luna, 2021). Collective mourning occurs when many people grief the loss of an individual simultaneously in each social milieu. Collective mourning prior to the diffusion of social media were mainly via public displays of collective grief. These typically take the form of grassroots temporary memorials as happens when people leave flowers, candles, and photos in a central public space (Wagoner & de Luna, 2021). However, these practices are changing since the diffusion of digital media technologies. It has been noted that advances in technology, particularly computer-mediated communication have changed “the death systems” by allowing online memorials, virtual cemeteries, and spaces for social support to occur instantaneously (Bingaman, 2022; Navon & Noy, 2021; Sofka et al., 2012, p. 6; Uwalaka, 2023a).

Studies in online mourning have outlined how the digital affordances of the internet created suitable spaces for remembering and memorializing the dead online (Cherasia, 2022; Jiwani, 2022; Uwalaka, 2021). It is increasingly becoming apparent that beyond this realm of mourning based on blood relationship, that there is a large hidden world of “connective mourning” where one mourns those that they do not have ties to or unrelated to but memorialized due to shared beliefs, and connective repertoires. In this study, “connective mourning” (Uwalaka, 2023b, p. 1) is conceptualized from the crisis network effect (Danowski, 1982; Danowski & Edison-Swift, 1985). Connective mourning is seen as a form of online mourning by a group of persons who hitherto did not have a tie or relationship with the deceased but only knew the deceased either through the nature of their death or the cause that the deceased is engaged in. It is slightly different with online collective mourning where the mourners use networked space to grief but are either related or know the deceased.

Crisis network effects have evaluated the impact of crisis such as natural disasters, sociopolitical upheavals such as assassinations, community controversies and individual’s information seeking and psychological function (Coleman, 1957; Danowski, 1982; Rogers & Sood, 1981; Schram, 1971). It has been noted that the “amount of communication increases” during a crisis (Danowski & Edison-Swift, 1985, p. 251). During crisis and uncertainty, messages became shorter, individual-levels networks became less interlocking, and that one large group was formed. This shows that when online collective mourning occurs, individuals have more reciprocal relationships on a dyadic level and that the network has low modularity. Such a network effect occurs when there is a shock that creates uncertainty in the system.

This study focuses on both the nature of the Queen’s death and her position in the society before her death. Thus, this paper explores how *X* users used the platform to mourn the death of Queen Elizabeth II.

Extant online mourning studies have either looked at the mourning practices of those that died in accidents, natural disasters, state brute like the killing of protesters (Uwalaka et al., 2023). Others have looked at mourning practices of celebrities who died young or abruptly such as Kobe Bryant (Bingaman, 2022). Some other social media mourning studies have evaluated the mourning practices of the passing of beloved and uncontroversial figures such as David the “Starman” and Stephen Hawkings (Akhther & Tetteh, 2023; Van den Bulck & Larsson, 2019). This study is needed to examine the mourning practices of people online on the death of a British monarch. While the Queen aged and died peacefully, the history of the institution that she headed for many decades is controversial and one that is often in the global discourse of racism and exploitation. The mourning of Queen Elizabeth II is unique as she was the first monarch to be mourned on social media, and that the discomfort of anti-monarchy as well as anti-racism movement are swelling. Will these variegated opinions and movements against the monarchy manifest in the mourning or will the populace engage in emotional rubbernecking (DeGroot, 2014).

This study extends current understanding of social media mourning and provides a unique perspective of online mourning practices of X users during the death of the first British monarch to be mourned online. This study improves the social media mourning literature in the area of parasocial relationships and effect they have on grief as well as disenfranchised grief.

## The Research Context

On 8 September 2022, Elizabeth II, Queen of the United Kingdom, the other Commonwealth realms, and the longest-reigning British Monarch, died at Balmoral Castle in Aberdeenshire, Scotland (BBC, 2022; Harrop, 2023; Soehardjo, 2022). She died at the age of 96. The Queen’s death started a cascading of event that was tagged, “Operation London Bridge” (Davies & Elgot, 2022; Harrop & Pearce, 2022; Leeuwen, 2022). This is a collection of plans and events including arrangements for the funeral of Queen Elizabeth II. The code, “London Bridge is down” is used by the Buckingham Palace to inform the Prime Minister and the Privy Council that the Queen has died.

According to Leeuwen (2022), the Foreign Office then notified the 15 other governments where the Queen was Head of State, as well as the 38 other commonwealth countries who belong to the Commonwealth. All these processes were observed upon Queen Elizabeth’s death. For example, Cabinet Ministers were informed, and flags on government buildings went to half-mast, and Britain’s national and regional parliaments adjourned (Leeuwen, 2022; Taylor & Parker, 2022). The announcement of the death of Queen Elizabeth II caused an unprecedented tight-knit community like response across the world. It plunged the United Kingdom and other Commonwealth nations into mourning. A mourning period was declared leading to the Queen’s liturgical state funeral (Harrop, 2023; Harrop & Pearce, 2022; Nichols, 2022).

Social media platforms were awash with condolence messages and grief. The Royal Family’s X handle @RoyalFamily posted the death of the Queen. The X announcement reads, “The Queen died peacefully at Balmoral this afternoon. The King and the Queen Consort will remain at Balmoral this evening and will return to London tomorrow” (BBC, 2022; Millson, 2022; The Royal Family, 2022). The Balmoral Castle and Estate posted the same announcement while Royal Central posted the same message with further context. The post announcing the death of the Queen received 2.4 million likes, 934,300 reposts and 85,500 replies. Most of the replies sent positive mourning comments such as “may she rest in eternal peace”, (Groves, 2022), and “RIP Her Majesty, Queen Elizabeth II” (Cowlin, 2022). The outpouring of love and solidarity at the passing of Queen Elizabeth II brings a new form of mourning to focus. This new form of mourning is what I attempt to conceptualize in this paper. The Queen’s death and announcement online not only was unprecedented but also created a networked mourning space that X users around the world used to grief on the death of the Queen. This study examines how X users used the platform to mourn and memorialize Queen Elizabeth II after her passing.

## Social Media as a Site of Mourning

Mourning is a means through which such persons display sorrow for permanently losing a relative to death (Gerrish & Bailey, 2020). Due to the way some people perceive death and dying, mourning the dead could take different forms and level. Consequently, variegated types of meanings could be deduced for the death of the person (Fernandez et al., 2011). Mourning has been referred to as “a process aimed at closure so that the mourners can get on with their lives, and nations are freed from the burdens of the past.” (Robben, 2023, p. 133). Some see it as either “understanding grief following loss” (Fernandez et al., 2011, p. 143) or “identification and validation of grief” (Marín-Cortés et al., 2023, p. 1). Collective mourning could depict show of respect to the deceased. Beside an instant response to the nature of death, collective mourning is also buttresses respect and honor to the deceased by many persons in the community (Wiederhold, 2017). Collective mourning could also be extended to those who died from certain disasters and collective mourning ensues as a means of showing certain for their loss (Bovero et al., 2020). This is even more so with the diffusion of social media platforms.

As social media innovate and diffuse, mourning practices are changing. For example, online mourning is increasingly becoming popular as mourners are becoming diverse and dispersed. Online collective mourning brings people from different geographical locations and cultures together into mourning a loved one or someone that hold certain meanings to the bereaved. This could for instance be “hidden protests expressed through multi-semantic mourning” (Cao et al., 2022, p. 159). Social media platforms such as X and Facebook allow for affective expressions of grief – underpinning a “networked sociality and articulating politics of opposition from the group-up” (Jiwani, 2022, p. 1).

The increasingly normative, ubiquity and affordances of these social media platforms allow for an increased expression of individualistic performances grounded in looser networks affinity. It does not matter whether these expressions and individualistic performances are primordial or situationally constructed in nature (Gotved, 2014; Jiwani, 2022; Morse, 2023a). These platforms “enable and empower those marginalized by traditional forms of grief to stay connected to the deceased” (Carroll & Landry, 2010, p. 1130; Morse & Birnhack, 2022). Through these networks, the mourners form collective memorial landscape which then build into a collection of enduring digital memories (Pasquali et al., 2022; Pennington, 2013; Veale, 2004).

Foundational studies around crisis network effects (Coleman, 1957; Danowski, 1982; Danowski & Edison-Swift, 1985) have evaluated the impact of crisis such as natural disasters, sociopolitical upheavals such as assassinations, community controversies and individual’s information seeking and psychological function (Coleman, 1957; Danowski, 1982; Rogers & Sood, 1981; Schram, 1971). Results have revealed that the “amount of communication increases” during a crisis (Danowski & Edison-Swift, 1985, p. 251). They further noted that during crisis and uncertainty, messages became shorter, individual-levels networks became less interlocking, and that one large group was formed. This foregoing shows that people email others that they have not emailed before a crisis. This connection helps the people to overcome the psychological traumatic experience.

Online mourning has been argued to facilitate “benevolent grief”, which is the use of grief for reintegration and recognition of the “other” as part of “us” (Morse, 2023b, p. 1302). This means that digital mourning rituals that are collectively, and synergically performed by the state and the media, advocate for the recognition of the marginalized other as belonging to the broader communities (Morse, 2023b). Online mourning helps mourners to identify and validate grief in their online communities. The deceased’s popularity is measured by the size of those following their death via social media buttons such as likes and emojis (Cherasia, 2022; Marín-Cortés et al., 2023; McCammon, 2022).

Extant social media mourning studies have shown that social media platforms are used to blend memorializing practices with existing practices and communication patterns for digital media (Brubaker & Hayes, 2011). Social media platforms have also enabled expansion – temporally, spatially and socially – of public mourning. Brubaker et al. (2013) note that rather than look at online practices as disruptions of traditional practices of grief and memorialization, that social media platforms should however be seen as new arena in which public mourning takes place. View social media platforms as new sites of mourning helps researchers uncover the relational continuity (Degroot, 2012) that online mourners have with the deceased. These social media platforms have been argued to serve two functions: sensemaking and continuing bonds or upholding relational continuity with the deceased.

Social media platforms have given mourners greater visibility and allow them to engage in mourning practices hitherto, they were unable to engage in. For example, social media platforms have been highlighted as the platforms of choice for the dissemination of news about the death of someone, preservation of the memory of the deceased and building a mourning community. It has been suggested that Facebook communication is both beneficial and challenging for the bereaved (Rossetto et al., 2015). This means that social media platforms can produce a coping paradox. This is evident when one understands that social media platforms can bring dying and grieving out of both private and public realms (Walter et al., 2012). This resonates with findings from other studies. For example, Moore et al. (2019) indicate a causal condition of social mourning such as sharing information with family and friends as well as initiating a dialog, discussing death with a broader mourning community, and commemorating and continuing connection to the deceased. This overexposure favored by social media mourning has been argued to question traditional ways of “living out” one’s grief, subjecting the living and the dead to a redefinition of concepts of time and space, and entail new forms of interactions (Dilmaç, 2018).

Social media mourning of celebrities takes a slightly different parasocial patterns. On a more general findings, Van den Bulck and Larsson (2019) confirms that X provides a virtual gathering of mourners who are looking for recognition of loss and expressions of support. On a specific note, online mourners of Stephen Hawking showed varied forms of mediatized emotional responses associated with parasocial grieving, such as sadness, shock, confusion, love and longing (Akhther & Tetteh, 2023). The mourners adopted some coping mechanisms including individualized tributes, reminiscing, memorializing and advocacy. It was therefore stated that mourners performed parasocial death rituals on X as a legitimate public space for mourning. Parasocial grieving such as sadness and shock were the most evident in the social media mourning of Kobe Bryant. Bingaman (2022) provides evidence to support the notion that emotional responses to grief like sadness dissipate over time while other emotional responses like love increase. Deaths from accidents like Kobe Bryant or suicide have more profound impact on the mourners and increase parasocial grieving rituals than natural death.

While many of the studies looked at mourning from accidents, depression, terrorism, and COVID-19, this study looks at mourning that was occasioned by the death of the longest serving Britain’s monarch. This study also content analyzed the posts of mourners who hitherto may not have known the deceased that they were mourning on X.

To achieve the above stated objective, this study sought to answer the following research questions:

1. What are the themes that emerged from posts and replies from X users around the world on the death and funeral of Queen Elizabeth II?
2. What do the diameter, density, reciprocity, centrality, and modularity measures reveal about the conversations around the death of Queen Elizabeth II?

## Method

### Data Collection

This study adopts a content analysis and social networking analysis approaches to data collection and analysis. This paper adopted a qualitative content analysis technique and specifically utilized social media network analytics. Using Netlytic (Malik et al., 2022; Meneses, 2019), I scraped 22,350 messages on X from the 21 of October, 2022 to 15 of December, 2022. After scraping the data, I downloaded the responses as an excel document into NVivo for qualitative data analysis. The goal was to download messages (posts, replies and reposts) concerning the Queen’s death. I wanted to understand the nature of the messages and condolence messages sent to the Royal Family on X. Further, I wanted to also understand the network measures and visualization to understand the type of crowds that were engaged on X during the death and funeral of the Queen. The hashtags, “The Queen” and “Queen Elizabeth II’s funeral” were used to collect the data for this study. This is because, there were no dedicated hashtag to mourn the queen and X users mourned by writing about the Queen. The second Hashtag was for her funeral.

### Data Analyses

The study analyzed posts and replies from the hashtags “The Queen” and “Queen Elizabeth II’s funeral” to determine the nature of conversations and crowds that used the hashtag, the number of posts and replies and the themes that emerged from the posts on how X users around the world mourned the death of Queen Elizabeth II. These two hashtags were the most used hashtags during the announcement of the death and funeral of the Queen. In this context, social media platforms offer many opportunities to conduct research on a wide range of topics and analysis of its content during mourning. It provides valuable insights irrespective of the researchers’ geographical location. Thus, this enables scholars to access data in diverse locations where field research could prove improbable. Also, *Facebook* and *X* are found to be two of the most promising sites for analyzing global debates on key issues due to the open environment of their data (Uwalaka, 2023a). While hashtags have been questioned as a sampling approach in big data analytics (Rafail, 2018), they are still one of the most used techniques to capture topic specific data in social media, particularly *X* and *Facebook*. Posts were scraped and some parts analyzed using Netlytic. In this analysis, keywords, were highlighted. Also, network properties were identified, analyzed and visualizations were observed and discussed. For the social network visualization, the study used the Distributive Recursive Layout (DrL) which is a “forced-directed graph layout, effective for visualizing large networks” (Meneses, 2019, p. 355; Pascual-Ferrá et al., 2022). In this layout, long edges are hidden to highlight clusters or communities of conversation. Clusters are groups of nodes that share a particular characteristic (Pascual-Ferrá et al., 2022). These communities appear on the graph as round or oval shapes (Santarossa et al., 2022; Suhaimi et al., 2021).

After analyzing the posts on Netlytic, the scraped posts and replies were downloaded from Netlytic and stored as a CVS file. The downloaded and stored dataset was then exported into NVivo (Crowley et al., 2002; Hilal & Alabri, 2013; Phillips & Lu, 2018) for further qualitative analysis.

I evaluated diameter, density, reciprocity, centralization, and modularity to understand the typology of the network. The diameter measures the longest distance between two users in the network, counted in the number of nodes or unique X user accounts (@name), that it takes to get from one participant to the other (Pascual-Ferrá et al., 2022). Density measures how close nodes are in a network. Reciprocity measures measure -way communication or how much nodes are talking to each other. Centralization measures the extent to which a few nodes dominate the conversation. Each node has a centrality measure: “indegree (based on times it has been mentioned or replied to)”, “outdegree (based on times it has mentioned replied to others)” and “total degree (the sum of both)” (Pascual-Ferrá et al., 2022, 563). The final network property is modularity. This measures the fragmentation of a network into distinct communities. For all these measures, values range from 0 (lowest) to 1 (highest). This means that a modularity values that is close to 1 indicate clear division between communities whereas, values less than .5 suggest that the communities overlap more; the network is more likely to consist of a core group of nodes (Pascual-Ferrá et al., 2022, 563). I also examined who was mentioned the most, who posted the most, and who were reposted the most to assess influence. In examining these different network properties, I was able to adjudge which among these network properties might affect the successful dissemination of mourning messages during the death and funeral of the Queen.

In the second part, the data were moved into qualitative software called NVivo. The data was saved in a CVS file format. This CVS file was then uploaded into NVivo. The software helped me to navigate the corpus of X posts and replies and highlighting related posts. This led to the broad categories that this study reports. The software helped retrieve code and build a conceptual framework that was handy at the theme development and meaning condensation stages. The codes drawn from the data were large in number. Consequently, the researchers submitted the codes to some form of analysis that would consolidate meaning. The researchers adopted thematic and meaning condensation approaches to make sense of the data. This approach “entails an abridgement of the meanings expressed by the interviewees into shorter formulations” (Kvale, 1996, p. 192).

My evaluation of the posts and replies yielded four broad themes: (i) condolences and sombre reflection, (ii) service and devotion (iii) accusation of racism and colonialism (iv) defence of the monarch.

## Results

### Results from Qualitative Content Analysis

The findings overall demonstrate that X users around the world used the platforms to mourn or criticize the Queen on her passing and funeral. Based on analysis of the data, I organized the findings into form themes. According to Table 1 below, these are (1) condolences; (2) service and devotion; (3) accusation of racism and colonialism; and (4) defense of the monarch.

**Table 1.** Broad Themes and Sample Broad Themes and Sample Tweets.

Themes	Sample comments
Condolences	• The Queen has died. We will not see her kind again. She will forever define servant leadership. • Our deepest and sincere thoughts are with the Royal Family. May the Queen rest in peace.
Service and devotion	• We will miss her humility, warmth, and her unwavering commitment to public service — in particular her service to Canada. A beautiful and dignified lady, and one of the best queens that ruled in England. • Forever in our hearts, thank you for everything, Your Majesty!
Accusation of racism and colonialism	• Racism has been outlawed in England since the 1960s, but many problems remain!! Why should black and brown people mourn. • Journalists are tasked with putting legacies into full context, so it is entirely appropriate to examine the Queen and her role in the devastating impact of continued colonialism.
Defence of the monarchy	• Don’t expect that of you but do expect common decency, respect for such a loss. If you cannot give that at this time, you are a disgraceful of a human being. • This is someone supposedly working to make the world better. I don’t think so. Wow.

### X – A Platform for Condolences, Grieving, and Mourning (Grievers)

The most prominent theme from the posts and replies that explains why X is central to mourning around the world is the notion that users have attached ideas of intensified mourning exchanges to the platform. The chance to mourn those who ordinarily one would not even know that they have passed is one of the reasons social media platforms such as X is popular. X users used the social media platform to grief and mourning the passing of the Queen.

After it was announced that the Queen has died, one X user posted, “may she rest in peace and her memory be a blessing”. Another posted, “We send our heartfelt condolences on behalf of the people of the Regional Municipality of Waterloo in Ontario, Canada.” Another noted, goodnight to the grandmother of our nation”. These posts and many others were mourning the Queen. These mourners ranged from just individuals to Heads of States. The kernel of these posts was to console the British people and to wish the Queen an eternal rest. This is evident with the number of “RIPs” that people posted on the day of the announcement and on the funeral day.

Another grieving and mourning aspect of this theme that was evident from the analyzed posts was that of sadness. X users displayed their sadness at the passing of the Queen. Although the queen was 96 years or age, people were still sad to hear that she had passed. This heartbrokenness and sadness could be attributed to the love and respect that mourners on X had for the Queen. One X user stated, “I am heartbroken hearing this news. May she RIP”. Another posted, “I’m so sad. I was 14 the day I heard the coronation on the radio. She has been my Queen too – all my life…”. Another X user said, “Queen Elizabeth’s death is saddening”. These posts buttress the sub-theme of sadness within the grief, mourning and condolence theme. These posts on this theme illustrate the respect and love that people the world over had for the Queen and the unabashed ways that they expressed the awe for the Queen was indeed touching. It reveals how much the Queen meant to people around the world. From the results, parasocial grieving rituals such as shock, sadness, reminiscing, and memorializing were most common responses of grief across X.

### Service and Devotion (Lauders)

This is another theme that was evident from the corpus of posts that was analyzed. X users provided anecdotal evidence to buttress their points that the Queen was one of the finest gentle ladies that has ever lived. X users around the world reposted Queen’s service oath to the people of Britain in 1947:

I declare before you all that my whole life whether it be long or short shall be devoted to your service and the service of our great imperial family to which we all belong.

The Queen pledged on her birthday in 1947 that she will dedicate her life to the service of her family and the United Kingdom. posts alluding to her service all praised her and often mentioned that she fulfilled her 1947 oath. X users noted that the Queen was “a loyal servant to the Crown” has a “lifetime of humility and loyal devotion”, “a wonderful lady who did so much for the country” and some noted that they will “miss her humility, warmth and her unwavering commitment to public service”. Other simply thanked her for her service. An X user posted, “thank you for your glorious service, Queen Elizabeth”. Another posted, “thank you for your service to the world” and yet another posted, “Godspeed, Elizabeth. Thank you for an amazing lifetime of service and dedication”.

These posts and many more show how appreciative X users were on the type of service that the Queen rendered. She was appreciated by even people who expressly noted that they were not royalists. Her service and dedication were transcendental and was larger than herself. People on X connected to Queen Elizabeth in death through her service to her people and the world. The posts evidently highlighted that people including those who were uncomfortable with the imperial history of the Royal Family, did respect the Queen for her sheer will to serve her people. This connectedness reverberated throughout the posts. The high regard that X users have for the Queen brought them together to grief a woman who they talked about as a servant and the embodiment of service and dedication. The posts demonstrate X users’ beliefs that the Queen was a servant leader. This genuineness shows that social media users were not engaging in an emotional rubbernecking (DeGroot, 2014).

### Accusations of Racism, Colonialism, and Defense of the Monarchy (Accusers and Defenders)

This last two themes are related. Hence, the fusion of their treatment here. While many X users praised the Queen for her tenacity to serve her people and all the good that she has done in her lifetime, some did post accusation at the Queen. These posts accused the Queen of benefitting from racism and colonialism. Some others accused her of killing Princess Diana and others pointed to the racism allegation levelled against the Royal Family by the Queen’s grandson, Harry and his wife, Meghan.

One of such posts, read “#reparations to all the countries that the #UK has exploited. #Goodriddance”. Another user posted, “real question for the ‘now is not the appropriate time to talk about negative impact of colonialism’ crowd: when is the appropriate time to talk about the negative impact of colonialism?”. Another user posted “telling the colonized how they should feel about their colonizers’ health and wellness is like telling my people that we ought to worship the confederacy”. Another user declared, “I heard the chief monarch of a thieving raping genocidal empire is finally dying. May her pain be excruciating”. These posts are critical of the monarchy. This critical stances against the monarchy led to outbursts against the Queen on her death. Having said this, a user posted a critical message against the Queen. The post reads:

At the moment, the thought of Diana and Meghan are keeping my eyes completely dry. I am surprised. I tend to weep even for personal enemies and structural oppressors, and the US media, entertainment and education has gilded her.

Another user posted about how the monarchy supervised a genocide that massacred her half of her family and concluded that she will only express disdain for the Queen and the monarchy. These posts are accusatory. They pointed to some historical wrongs that they Royal Family and the British Government have supervised. Those posting this anger filled response to the Queen expressed their derision at the monarchy and saw the Queen as its ultimate symbol.

These posts showed the contempt of those that posted it on X brought responses from the defenders of the monarchy. Many of the defenders called out those that posted critical message against the Queen and the Royal Family. They called posts against the Queen “uncouth”, “unbecoming” and “foolish”. They noted that it was the Queen that instituted colonialism and many of the defenders explained that it was in Queen’s reign imperialism was curtailed and the fight against racism initiated.

One of the defenders in response to one of the critical messages posted, “you vile disgusting moron”. Another noted, “you speak of someone who just passed with such a vile and disdaining comment”. The reactions of the defenders of the monarchy were that posting messages that were critical of the Queen at her passing was disrespectful and unbecoming. The defenders passionate defense of the crown points to the love and respect that a lot of people have for the Queen.

### Results from Social Networking Analysis

**Figure 1.**
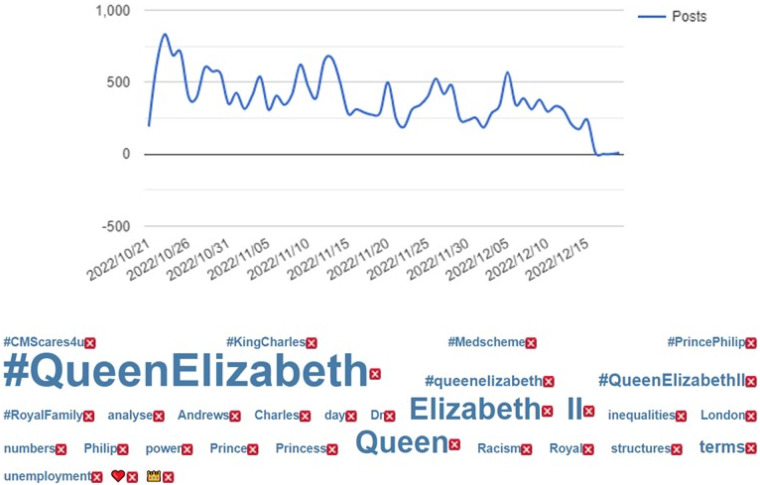
Word cloud and data collection dates.

**Figure 2.**
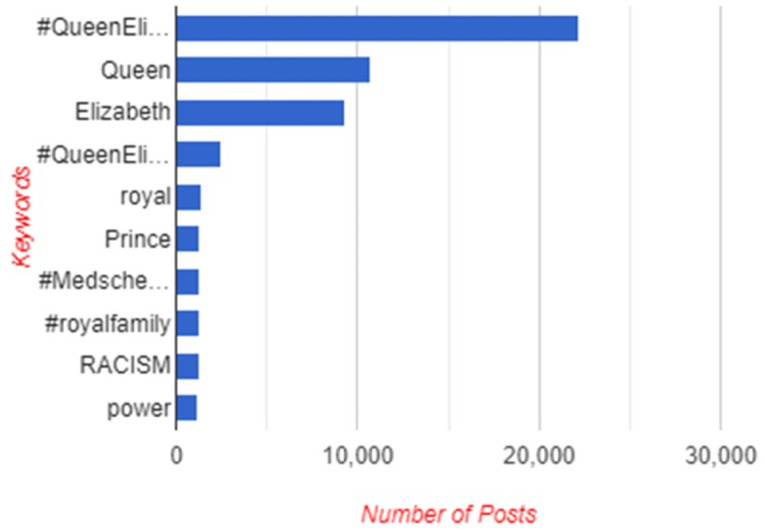
Ten most frequently used keywords.

Figures 1 and 2 below highlights ten keywords that were prominent in the posts and replies from X users around the world. The keyword that was most used in posts is “#QueenElizabeth”. It was mentioned about 22,251 times from this study’s data. This is followed by, “Queen” (16,766), “Elizabeth” (9388), “QueenElizabethII” (2453), “Royal” (1364), “Prince” (1302), “Medscheme” (1293), “RoyalFamily” (1288), “Racism” (1247), and “Po r” (1212). The “prince” keyword refers to how the X users reacted to Prince Charles becoming the King of Britain. Also, the numerous keywords relating to the Queen were words and expressions used to mourn and grief for the death of the Queen. These numbers and themes show that the Queen was front and center in the online discourses around her death. The numbers further reveal racism was raised as an issue in the monarchy history in X discursive arena. This result shows how frequent X users used parasocial mourning rituals such as sadness, reminiscing and memorialization in their mourning of the Queen.

**Figure 3.**
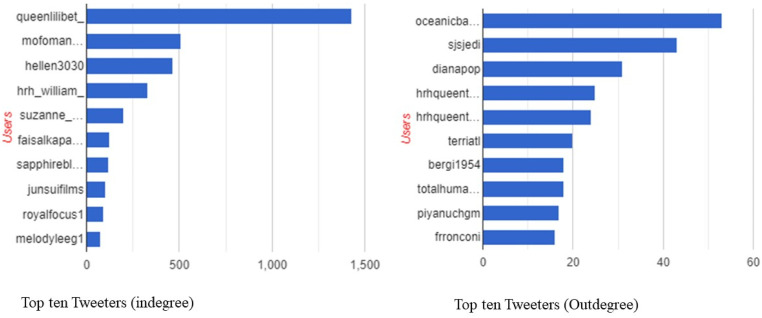
Top ten Tweeters (indegree and outdegree).

Furthermore, Figure 3 below shows the top ten posters (indegree and outdegree) during the death and funeral of Queen Elizabeth II on X. In the indegree section, that is posters who were reposted, quoted, and replied the most, @Queenlilibet the highest number of inwards directed about the passing of the Queen. She was followed by @mofoman, @hellen3030, @hrh_William, etc. For outdegree, that is those who posted the most, they are @Occeanicbaby, @Sjsjedi, @dinapopo, hrhQueentammy etc.

**Figure 4.**
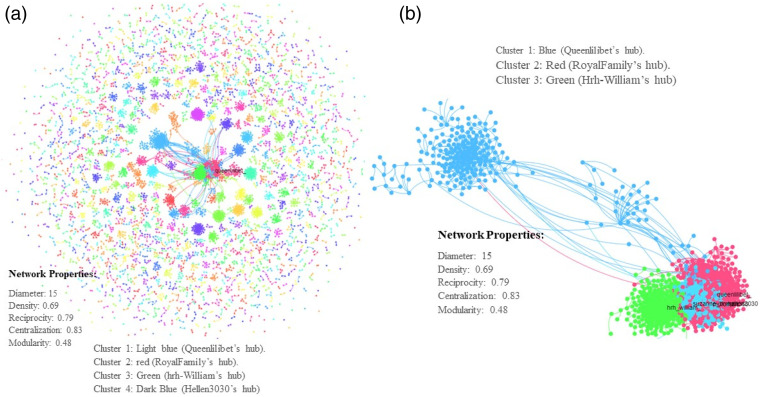
Network visualization.

**Figure 5.**
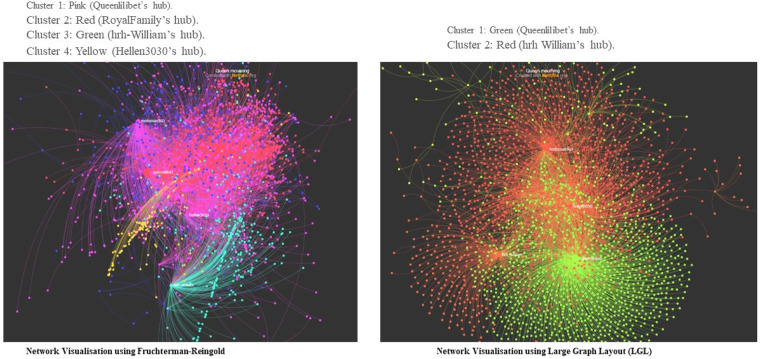
Network visualization (Fruchterman-Reingold and LGL).

In Figures 4 and 5 below, the diameter is 15. In network properties, this means that the longest distance between two users in the network is 15. This is counted in the number of nodes or unique X user accounts that it takes to get from one participant to another. Density measures how close nodes are in the network. In this Figure, the density is .69, reciprocity = .79, centralization = .83 and modularity = .48. Reciprocity measures two-way communication or how much nodes are talking to each other. Centralization measures the extent to which a few nodes dominate the conversations. Each node has a centrality measure – indegree (based on times it has been mentioned or replied to) and outdegree (based on the number of times it has mentioned or replied to others). The sum of both is labelled “total degree”. Modularity measures fragmentation of networks into distinct communities. For all these measures, values range from 0 (lowest) to 1 (highest). Based on this, the modularity of the graph is less than .5. This suggests that as the communities “overlap more; the network is more likely to consist of a great core group of nodes” (Pascual-Ferrá et al., 2022, 563).

Figures 4 and 5 show that the conversations in these Figures are centralized, has high reciprocity – meaning that participants were talking amongst themselves and are a close-knit and homophilous network. This is because of the moderate value of the diameter and the high density of the conversations. The point here is that although the Royal Family did not engage with those who responded to the announcement about the Queen’s death, that X users did in fact communicate with one another.

The network pattern is alike when the I visualized the network outputs in all layouts. The two Chats in Figure 4 are DrL outputs while Figure 5 shows the outputs of Fruchterman-Reingold, a popular forced based algorithm that works best when networks have 1000 or less nodes (Fruchterman & Reingold, 1991), and Large Graph Layout (LGL), which is used to visualize large networks while avoiding hairballs (Adai et al., 2004).

One of the most influential network actors in the discourse about the death and funeral of the Queen is an X user @Queenlilibet. A look at Figure 4 reveals that @Queenlilibet, who is the originator of cluster 1 (light blue) in chart “A”, and “blue” in chart “B” of the network visualizations. @RoyalFamily, who is in red and the originator of cluster 2 in both charts, @hrh_William, who is green in both charts and @hellen3030, who is the originator of cluster 4 and dark blue were all influential in the network. Figure 5 shows how influential @Queenlilibet (pink in the Fruchterman-Reingold output and green in LGL output) is in the network. The account @hrh_William had a pronounce influence in the network analyses.

These figures show the building of networks from these network actors and their connections to others based on some common form of interaction (ties). The charts in Figures 4 and 5 show that as X users mourn the Queen, that they developed on an individual level, more reciprocal relationships. This reciprocal relationship on a dyadic level, which also is demonstrated with the networks low modularity, occurs when there is a shock that creates uncertainty in the system.

## Discussion

This study demonstrates that online mourners on Xers used the platform to mourn the death of Queen Elizabeth II. A broad look and interrogation of the coordinated way X users mourned the passing of the Queen has opened a new window into how scholars can begin to study and appreciate the empowering nature of social media platforms particularly during crisis, such as mourning. The qualitative content analysis uncovered that those who posts about the death and funeral of the Queen were either grievers, lauders, accusers, or defenders.

The *Grievers* were those that were saddened about the death of the Queen and showed through their posts how sad that they were of the passing of the Queen. The grievers posted their condolence messages and often prayed for the Queen’s souls to rest in peace. The Grievers used parasocial mourning rituals such as sadness, shock, reminiscing and memorialization to mourn the Queen. The result of this study shows that X users used the platform to expand the spatial and social space for mourning. They have mainstreamed X as a public space to mourn. Through their posts and replies, X users upheld their relational continuity with the Queen (Degroot, 2012).

The *Lauders* are also people that love and cherishes the Queen. This cohort specifically love the Queen and extoll her devotion and service to her people. The lauders posted “thank you” messages to the Queen. They posted the Queen’s 1946 service oath to her people. They uniformly agreed in their posts that the Queen upheld her service oath and served the people of Britain with distinction. The lauders feted the Queen and appreciated her devotion to her people. The *Accusers* are dissimilar to the other three cohorts in that they expressed anger towards the monarchy. They hate the monarch and accused her of racism and “white supremacy”. They anchored their grievance posts in three points, colonialism, racism against Meghan Markle – the wife of Prince Harry, and the treatment of Princess Diana. Those who posted the impact of British imperialism accused the Queen of being a beneficiary of an inhuman and racists system. While those who complained about the treatment of Duchess Meghan Markle accused the monarch and her family of maintaining a racist posture. Other accusers blamed the Queen for Princess Diana’s death. They frowned at the treatment of Princess Diana, and her untimely death.

*Defenders* posted justifications for some of the accusations levelled against the Queen and the monarchy. They defended the Queen by absolving her of blame. They argued that the Queen was not part of or benefitted from colonization. The defenders posted about the Queen’s involvement in decolonization and her efforts to mend some of the ills of her predecessors. This result shows that the grievers, lauders, and defenders mourned the Queen as a way to show their respect and to honor her (Wiederhold, 2017) while the accusers did not show her such respect. The way the Queen lived her life and the institution that she represent showed how she was mourned and who did the mourning on X (Fernandez et al., 2011; Stevenson et al., 2016).

This study shows that social media platforms such as X are increasingly used to disseminate news of the passing of a member of the community, beginning of dialog, discussing death with a broader mourning community, and used to perform certain parasocial mourning rituals (Moore et al., 2019; Rossetto et al., 2015). The *Grievers* and *Lauders* used the posts on X to mainly perform parasocial mourning rituals, to disseminate the news about the death of the Queen, and to discuss the death of the Queen with a broader mourning community. However, the *Accusers* and *Defenders* used their posts and replies on X to start a dialogue around racism and the involvement and lack of involvement of the Queen. The nature of the dialogues in this study shows how online mourning could disintegrate into the abuse of the deceased. The lack of control could lead to the overexposure of the actions of the deceased that may not be in line with the society’s expectations. This could sully the memory and memorialization of the deceased. In the case of Queen Elizabeth II, she had capable Defenders who competently nullified the accusations levelled against her.

The network visualization in Figures 4 and 5 above show a tight crowd, interconnected by issues that the online mourners are deliberating. What this illustrates is that digital mourners on X are switched on and engage with one another in a substantive way. A closer look at what they are deliberating on X, shows topics ranging from the Queen’s lifetime of service, her achievements, condolences, and accusation of racism. The valence of the posts is critical and robust. The closeness and interconnectedness of the networks show that this was not a polarized crowd but a tight crowd where all participants were talking to one another and engaging in substantive topics – mourning the Queen, extolling her dedication and devotion to her people, accusation of colonialism and racism and defense of her record.

This study demonstrates that decentralized technologies result in loosely inter-connected, interpersonal networks which create outcomes that resemble collective mourning, yet without having solid ties with the deceased. Finding from this study confirms the result of Bennett and Segerberg (2012) which uncovered that personal networks’ diversity provided a far stronger explanation for the predominant reliance on digital media than simple associations with the Royal Family. This study reveals that digital media’s affordances, replicability, scalability and searchability created apt digital environment for mourning, remembering, and memorializing Queen Elizabeth II, her life, and achievements. This means that beyond this realm of relatedness to mourn the Queen, there is a large hidden world of connective mourning (Uwalaka, 2023b; Uwalaka et al., 2023), where one mourns those that, they do not have ties to or are unrelated to but memorialized due to shared beliefs and connective repertoires. The study further show that digital mourners are embracing more expressive styles of mourning defined around social media platforms such as X and peer content sharing. Connective mourning such the mourning of the Queen on X shows that there are growing opportunities to have a shared grief in the society even for people who are unrelated to the deceased by blood, family, or country. However, the mourners are only connected to the deceased via their shared humanity.

This study uncovers that those grieving online acted as mediators within the mourning community as they mourned on an individual basis and yet, contributed towards the collective mourning goals. Although, these mourners acted on an individual basis such as individualized tributes (Akhther & Tetteh, 2023), the common concern of respecting the service of the Queen brought them together. This togetherness of grief was not built through blood ties or strong and thick relations, but through anger and common concern. However, because the grief was situated around a common concern (mourning the death of the Queen), this stimulated feeling of collective grief or “benevolent grief” and connective memory which provided a form of collective memorial landscape, helped to build an enduring memory of the deceased (Hoskins, 2011, 2016; Morse, 2023b; Pennington, 2013). Online mourners’ ability to stimulate commonality via decentralized and loose networks while allowing for solidarity building during mourning demonstrates the personalization of mourning and online connectedness of humanity. These networks help identify and validate grief in online groups (Marín-Cortés et al., 2023).

Beside the emergence of the themes, this study also showed the networked relationship among digital mourners. Figures 4 and 5 above showed a tight crowd where information disseminations revolve around a few visible participants who then are placed in an opinion leadership position. The nature of this network showed that the topic under discussion was important and interesting. This demonstrates that mourners used the organizing tools X to mourn and laud the Queen for her service. The high centrality, high density of reciprocity and low modularity lead to fast diffusion of information and promise to encourage widespread adoption of the communique proposed by the mourners around the world.

This study reveals that when collective mourning occurs online, individuals have more reciprocal relationships on a dyadic level and that the network has low modularity. Such a network effect occurs when there is a shock that creates uncertainty in the system. This is related to Danowski and Edison-Swift (1985) “crisis network effects” findings. Danowski and his colleague noticed that people emailed others that they have noted emailed before during a crisis and that the messages were shorter and “one large user group formed” (Danowski & Edison-Swift, 1985, p. 251). Also, earlier results have shown that death from situations such as suicide had more profound impact and emotional stress on the mourners (Lester, 2012). This is akin to the network visualization in Figures 4 and 5 where the mourning communities were tight-knitted and appeared to be grieving together. It shows that when a community experiences collective mourning, individuals may seek out others, who are also affected by the loss for comfort, understanding, and emotional support. This process can lead to the forming of new relationships or strengthening existing ones, resulting in a more interconnected and reciprocal network at the dyadic level. The crisis network effects theory suggests that a similar pattern occurs with crises of different kinds (Coleman, 1957; Danowski, 1982; Rogers & Sood, 1981). Connective mourning creates a sense of shared vulnerability, like when other crises occur, leading individuals to empathize with others who are also grieving. This empathy may encourage people to reach out to others they have not frequently communicated with before the crisis or shocking incident, thus fostering new connections and reducing network modularity.

## Limitations of the Study

This study has some limitations. Although the data for this study was from 67,678 posts and replies about the about the death of Queen Elizabeth II, I acknowledge that the study is limited in the number of posts that were collected for the purpose of this study. For example, this study only collected data from two hashtags among the many that were used during the death and funeral of the Queen. Also, the categories that Netlytic showed were in single word format that needed a human subjective compilation. The categories used in the qualitative analysis were chosen by me and not validated by previous studies. Having said all these, I took steps such as using multiple analytic approaches to ameliorate these limitations.

## Conclusion

The study illustrates that digital media acts as mourning and solidarity platforms where mourners plan, coordinate and mourn their fallen compatriot and leader. Results show that mourners on X communicated their grief and shared their condolence on the death of the Queen. The study further shows that X helps mourners to organize memorials to not only sustain the grieving but also to praise, accuse and defend the deceased.

This study demonstrates that in times of crisis or uncertainty, people search for information to make sense of the situation and reduce feelings of confusion or helplessness. This can lead individuals to connect with others who may possess relevant knowledge or experiences, even if this is the first time, they have communicated with them. This explains why the network becomes less modular. The findings from this study suggest that when a community experiences a shock that creates uncertainty, such as the death of a Queen, its members bands together to cope and rebuild their sense of security. This collective coping fosters new connections and strengthen existing ones as individuals work together to make sense of the uncertain environment and reduce stress.

A higher-order construct encompassing the phenomena in this study is an emotional disturbance producing a significant increase in negative emotions. Emotional disturbances can result from various crises, including the death of a monarch as was the case in this study or other traumatic events, leading to heightened stress levels. To reestablish baseline functioning, individuals need to alleviate this stress. One effective way to reduce this type of stress is through the sharing of emotions openly and without filters. Connecting with new online users facilitated a deeper sharing of emotional responses. Established social relationships might not be optimized for unbridled emotional expression, as it can threaten perceived social acceptance and may not provide the support needed in times of crisis. This is why there was a preference for connective engagement as the mourners were able to share high vulnerability more readily with strangers or less familiar contacts. Sharing intense emotions with new connections can lead to more of a sense of release and shared relaxation response, ultimately proving more effective in coping with emotional disturbances.

The study shows that the need for stress reduction and emotional support drives individuals to reach out to new contacts, leading to more reciprocal relationships at the dyadic level which then decreases network modularity. What this means is that the death of someone beloved leads to greater emotional stress and that this stress motivates mourners to communicate with one another as a support system.
